# Scanning tunnelling spectroscopy of superconductivity on surfaces of LiTi_2_O_4_(111) thin films

**DOI:** 10.1038/ncomms15975

**Published:** 2017-07-03

**Authors:** Yoshinori Okada, Yasunobu Ando, Ryota Shimizu, Emi Minamitani, Susumu Shiraki, Satoshi Watanabe, Taro Hitosugi

**Affiliations:** 1 Advanced Institute for Materials Research, Tohoku University, Sendai 980-8577, Japan; 2 Department of Materials Engineering, The University of Tokyo, Tokyo 113-8656, Japan; 3 Research Center for Computational Design and Advanced Functional Materials, National institute for Advanced Science and Technology, Tsukuba, Ibaraki 305-8568, Japan; 4 School of Materials and Chemical Technology, Tokyo Institute of Technology, Tokyo 152-8552, Japan

## Abstract

Unique superconductivity at surfaces/interfaces, as exemplified by LaAlO_3_/SrTiO_3_ interfaces, and the high transition temperature in ultrathin FeSe films, have triggered intense debates on how superconductivity is affected in atomic and electronic reconstructions. The surface of superconducting cubic spinel oxide LiTi_2_O_4_ is another interesting system because its inherent surface electronic and atomic reconstructions add complexity to superconducting properties. Investigations of such surfaces are hampered by the lack of single crystals or high-quality thin films. Here, using low-temperature scanning tunnelling microscopy and spectroscopy, we report an unexpected small superconducting energy gap and a long coherence length on the surface of LiTi_2_O_4_(111) epitaxial thin films. Furthermore, we find that a pseudogap opening at the Fermi energy modifies the surface superconductivity. Our results open an avenue for exploring anomalous superconductivity on the surface of cubic transition-metal oxides, where the electronic states are spontaneously modulated involving rich many-body interactions.

## Abstract (web summary)

Atomic and electronic reconstructions at surfaces/interfaces bring about various emergent phenomena. Here, Okada *et al*. report an unexpected superconducting gap and a long coherence length on the surface of LiTi_2_O_4_ (111) thin films.

Recent advances in the atomic-scale synthesis and characterization of two-dimensional superconductors have kindled significant interest in their exotic electronic, orbital and magnetic structures^1^,^2^,^3^,^4^,^5^,^6^. In addition to the ultrathin superconducting films on substrates^7^,^8^,^9^,^10^,^11^,^12^,^13^,^14^,^15^,^16^,^17^,^18^,^19^,^20^, surfaces of cubic-structure superconducting transition-metal oxides provide another interesting platform, because the spontaneous electronic and atomic reconstructions on surfaces are expected to modify superconductivity. A particularly interesting system is the spinel oxide (*AB*_2_O_4_) superconductor. In this system, the cubic pyrochlore sublattice of *B*-atoms provides large degeneracy (frustration) of charge, spin and orbital states in bulk^21^,^22^, and a prominent degeneracy lifting at the surface is expected to lead rich electronic states on the surface. None of the previous studies, however, has revealed the electronic signature of modulated superconductivity on their surfaces.

Lithium titanate, LiTi_2_O_4_, is the only oxide superconductor with spinel structure^23^,^24^,^25^,^26^, and exhibits the highest superconducting transition temperature (*T*_c_) with ∼13.7 K of any spinel superconductors^27^,^28^,^29^,^30^,^31^,^32^. It is known as a 3*d*^0.5^ metallic system (one half of an electron per Ti atom resides in the 3*d* states), and medium-coupling Bardeen–Cooper–Schrieffer (BCS) superconductivity with *s*-wave pairing symmetry has been proposed^33^,^34^,^35^,^36^. Recent transport data with epitaxial films showed an angle-dependent anomalous magnetoresistance, which is possibly related to a spin and orbital fluctuation effect^22^. On the basis of this data, the similarity between LiTi_2_O_4_ and high *T*_c_ cuprates has been discussed^22^. Although superconducting properties of bulk LiTi_2_O_4_ have been investigated extensively, studies of the superconductivity at LiTi_2_O_4_ surfaces have been hindered by the lack of single crystals and high-quality thin films. Recently, high-quality epitaxial LiTi_2_O_4_ thin films were successfully grown using pulsed laser deposition (PLD)^37^,^38^,^39^; however, investigations of the superconductivity on LiTi_2_O_4_ surfaces remain unexplored. Recently, we have developed a scanning tunnelling microscope (STM) combined with a PLD system^40^, and *in situ* investigation of the LiTi_2_O_4_ surface without exposing to air has became possible.

Here we report modified superconductivity at the surfaces of LiTi_2_O_4_(111) epitaxial thin films, using STM/scanning tunnelling spectroscopy (STM/STS) and first-principles density functional theory (DFT) calculations. From the atomic-scale observations of superconductivity, we present measurements of the unexpected small superconducting energy gap *Δ* and a long coherence length *ξ* values. Furthermore, we found that a superconducting gap exists on large energy scale pseudogap states. These results provide the spectroscopic evidence of spontaneous superconducting modification on the surface of cubic transition-metal oxides, paving an interesting path to explore exotic superconductivity involving rich interactions.

## Results

### Preparation of higher *T*_c_ sample with flat surface

**Figure 1:**
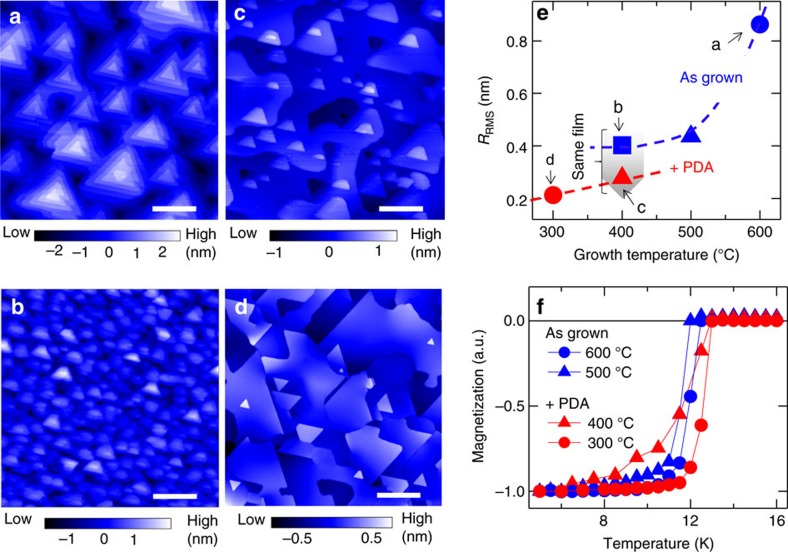
Surface topographies and superconducting critical temperatures. (**a**,**b**) STM topographic images of as-deposited thin film at substrate temperature of 600 °C (**a**) and 400 °C (**b**). (**c**,**d**) STM images after post-deposition annealing (PDA) for films deposited at 400 °C (**c**) and 300 °C (**d**). Note that **b**,**c** are taken with using the same film. **a**–**c** are obtained at 77 K and **d** is obtained at 4.2 K (all the STM images were observed at a sample-bias voltage of +300 mV and a tunnelling current is about 10 pA). Scale bar, 80 nm (**a**–**d**). (**e**) Growth temperature dependence of root mean square of surface roughness (*R*_RMS_) values: as-grown samples (blue symbols) and after PDA (red symbols). The value of *R*_RMS_ is evaluated from topographic images observed at a sample-bias voltage of +300 mV and a tunnelling current of 10 pA (scan area of 400 nm). (**f**) Temperature dependence of the field-cooled dc magnetic susceptibility for the LiTi_2_O_4_ films in a magnetic field of 50 Oe, which was applied parallel to the (111) plane. Clear diamagnetism is observed.

We found that low-temperature film deposition using PLD followed by post-deposition annealing increases *T*_c_ up to 13 K, together with improved surface flatness. We first compare the STM images of the films grown at substrate temperature *T*_s_ of 600 °C (Fig. 1a) and 400 °C (Fig. 1b). The former temperature is the typical growth temperature reported in the previous studies^22^,^37^,^38^,^39^. While many triangular-shaped islands are observed on both surfaces, the height of the islands is much smaller in the latter film, indicating that the surface roughness is strongly dependent on the growth temperatures. As the *T*_s_ is reduced from 600 to 400 °C, the root mean square (RMS) value of surface roughness, *R*_RMS_, improved from 0.86 (Fig. 1a) to 0.40 nm (Fig. 1b). To further improve the surface roughness, we investigated the post-deposition annealing effect on the film grown at *T*_s_=400 °C; thin films were annealed at 600 °C in ultrahigh vacuum for an hour. Expectedly, the film became flatter to *R*_RMS_=0.28 nm (Fig. 1c). Furthermore, this *R*_RMS_ value was improved to 0.21 nm for the film deposited at *T*_s_=300 °C followed by the above-mentioned annealing (Fig. 1d and Supplementary Fig. 1).

Importantly, this annealing process increases *T*_c_ values (Fig. 1f), in addition to the flattening of the surfaces. While the *T*_c_ values of the films grown at *T*_s_=500 and 600 °C show 12.0 K and 12.5 K, respectively, the *T*_c_ increases up to 13.0 K for the films after annealing (red curves in Fig. 1f). The *T*_c_ value of ∼13 K is one of the highest values among reported values for LiTi_2_O_4_ epitaxial thin films^22^,^37^,^38^,^39^. We confirmed from X-ray diffraction that the films exhibit rocking-curve full-width at half-maximum of 0.36° for 444 peak, and that the lattice is fully relaxed from SrTiO_3_(111) substrate (Supplementary Fig. 2). The above results show that the quality of the sample is quite high. In the following investigations, we focus on the films deposited at *T*_s_=300 °C followed by the post-deposition annealing in vacuum at 600 °C.

### Atomic-scale order on LiTi_2_O_4_(111) film surface

**Figure 2:**
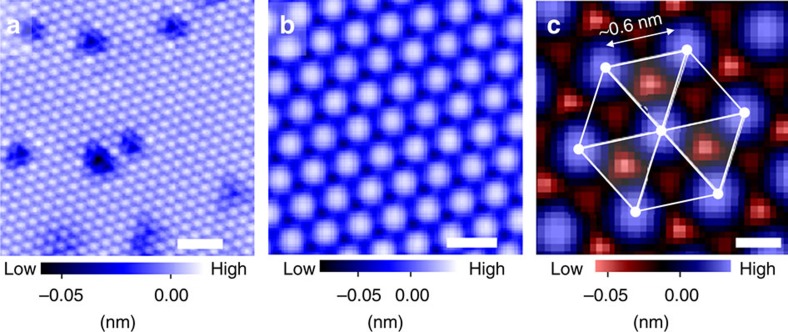
Typical topographic images on a terrace. (**a**) Filled-state STM image of LiTi_2_O_4_(111) surface (11.6 nm × 11.6 nm, sample-bias voltage *V*_s_ of −900 mV, tunnelling current *I*_set_ of 30 pA). (**b**) Empty-state STM image (4 nm × 4 nm, *V*_s_=+30 mV, *I*_set_=30 pA). (**c**) Zoomed-up image (1.7 nm × 1.7 nm, *V*_s_=+30 mV, *I*_set_=30 pA) of **b**. The image shows three-fold symmetry representing the spinel crystal structure. Scale bars, 2 nm (**a**), 0.8 nm (**b**) and 0.3 nm (**c**).

We observed, on the atomic level, a well-ordered triangular lattices and defects. Figure 2a shows the wide-area STM image of the LiTi_2_O_4_ surface, where flat terraces and dark spots with peculiar defect shapes were observed. In the close-up image (Fig. 2c), strong topographic corrugations (shown in blue) are observed together with weak topographic corrugations (shown in red), displaying three-fold symmetry. This is consistent with the inherent three-fold rotational symmetry along the [111] axis in a spinel system. The unit cell size was found to be ∼0.6 nm (Fig. 2c), which was independent of the location and sample-bias voltages *V*_s_. This size well matches that of the unit cell of LiTi_2_O_4_ bulk (111) plane. We concluded that the protrusions of the triangular lattice originate from Ti atoms on the surface, as discussed later together with the nature of the defects (dark spots).

### Superconductivity in tunnelling spectra measurements

**Figure 3:**
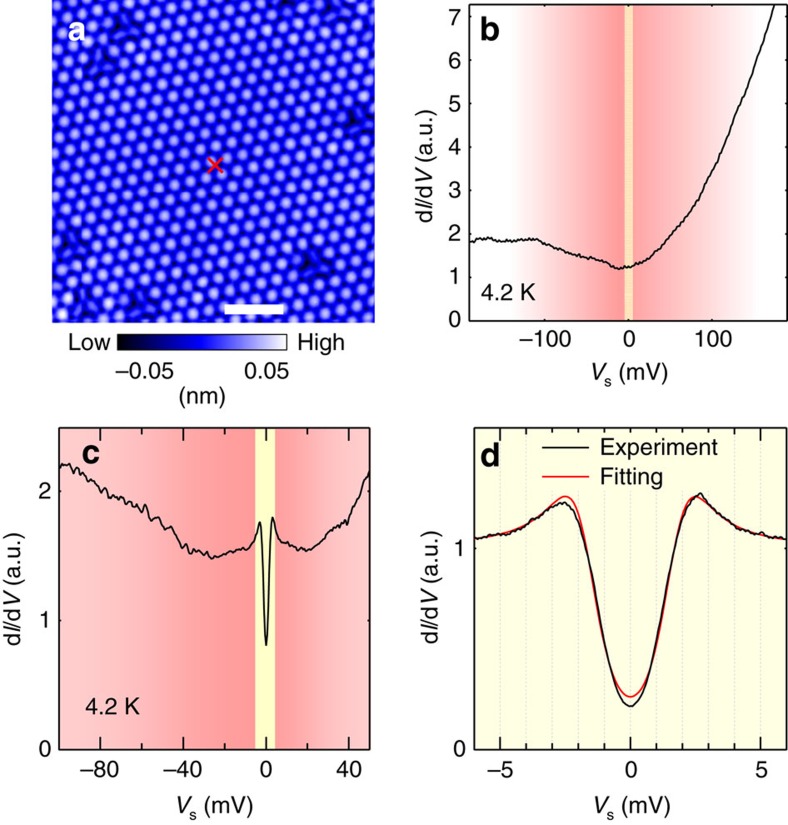
Tunnelling spectra obtained away from defects. (**a**) A topographic image obtained with a sample-bias voltage *V*_s_ of +30 mV and a tunnelling current of 30 pA. Scale bar, 2 nm. The red cross in **a** indicates the position where the spectra shown in **b**–**d** were obtained. (**b**,**c**) Tunnelling spectrum (d*I*/d*V*) obtained at a wide energy region (*V*_s_ of −190 mV to +190 mV and −100 mV to +50 mV, for **a**,**b**, respectively.) (**c**) High-resolution tunnelling spectrum for *V*_s_ between ±6 mV near the Fermi energy. Experimental curve (black dots) and fitted curve (red line) are shown in the same figure. See main body for the details of the fitting procedure. The energy windows of numerical derivative to obtain conductance spectrum were 15, 1.5 and 0.3 mV for **a**–**c**, respectively. The yellow region represents the *V*_s_ range of −6 to 6 mV. All the images and spectra were obtained at 4.2 K.

In addition to the atomic-scale corrugations, we observed clear signature of superconductivity in tunnelling spectra measurements at 4.2 K. The wide- and narrow-energy-range spectra were obtained at the area far from defects (see cross in Fig. 3a). To evaluate the superconducting gap *Δ* quantitatively, we analysed tunnelling spectra (Fig. 3b–d) using the Dynes formula^41^ convoluted with an energy derivative of the Fermi–Dirac function *F*.


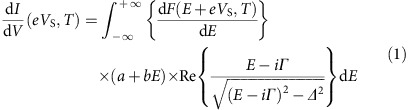


Here we assume that the normal-state density of states (DOS) near *E*_F_ has a linear dependence with energy *E*, expressed as (a+b*E*), and *Γ* is the spectral broadening factor. The fitting parameters a, b, *Δ* and *Γ* are determined with fixed temperature *T*=4.2 K. We fitted our spectra (Fig. 3d) with a single-component isotropic pairing gap, and we obtained *Δ*=1.716±0.004 meV and *Γ*=0.321±0.004 meV. According to the BCS gap function, assuming the same *T*_c_ for surfaces and bulk (13 K), the obtained *Δ* value can be regarded as a gap value in the *T*=0 K limit. With using bulk *T*_c_, the obtained 2*Δ*/*k*_B_*T*_c_ value of 3.0 is unexpectedly small. In contrast, the 2*Δ*/*k*_B_*T*_c_ values reported in polycrystalline LiTi_2_O_4_ samples range from 3.5 to 4.0 in point-contact spectroscopy^33^,^34^ and Andreev reflection^36^. A recent report using epitaxial LiTi_2_O_4_ thin films claims 2*Δ*/*k*_B_*T*_c_=4.07 from point-contact spectroscopy^22^, and thus the obtained value of 3.0 is much smaller than that of all the previous reports. Furthermore, the present value is even smaller than that for the weak coupling limit for *s*-wave BCS superconductivity of 3.52. We discuss later the possible origins of this unexpectedly small 2*Δ*/*k*_B_*T*_c_.

### Coherence length on the surface

**Figure 4:**
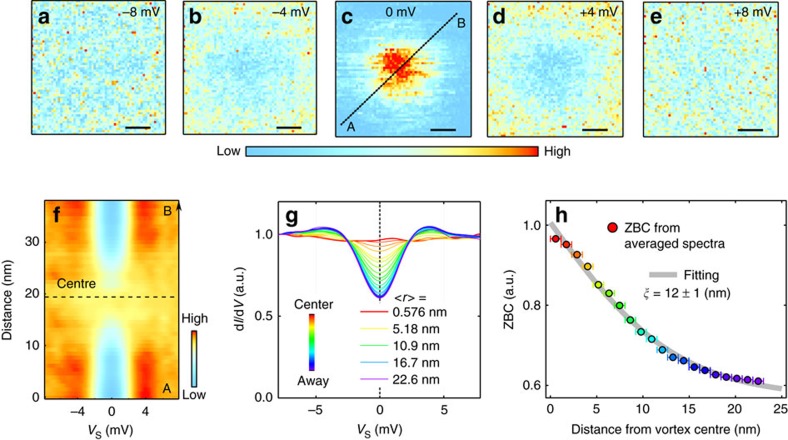
Spectral evolution around a vortex core. A single vortex core is investigated at the temperature of 4.2 K with applying a magnetic field of 1.5 T normal to the surface. (**a**–**e**) Conductance mappings around an isolated single vortex core with various sample-bias voltages. A STM tip is stabilized at a tunnelling current of 30 pA and a sample-bias voltage of −10 mV. The energy window of numerical derivative to obtain conductance spectrum is 1 mV. Scale bars, 6 nm (**a**–**e**). (**f**) Spatial evolution of tunnelling spectra across the vortex centre (the line is shown in **c**). (**g**) Averaged spectra as a function of distance from vortex centre <*r*>. (**h**) Zero-bias conductance (ZBC) obtained from **g**. Coherence length of ∼12 nm is obtained by fitting the ZBC (see main text for details). A total 3,600 spectra (60 × 60) were taken with equal spacing in the region (**a**–**e**). We classified the area of Fig. 4a–e into 20 regions based on the distance from vortex centre (see also Supplementary Fig. 3). Interval of <*r*>, which is 5.758 nm, is set as error bars for *x* axis in **h**.

To further study the superconductivity on the surface, we investigated the value of *ξ* from the electronic structures around a magnetic vortex core. We first analysed the *V*_s_ dependent conductance (d*I*/d*V*) map around a single vortex core by applying an external magnetic field of 1.5 T perpendicular to the surface at 4.2 K (Fig. 4a–e). At *V*_s_=−8 mV and +8 mV, we observed uniform conductance over the scanned region (Fig. 4a,e), whereas conductance values were depressed around the centre of images at *V*_s_=−4 mV and +4 mV (Fig. 4b,d). This depressed conductance is a consequence of suppressed coherence peaks. In contrast, the conductance map at *V*_s_=0 mV clearly represents enhanced conductance in the centre region (Fig. 4c). This enhanced zero-bias conductance around the centre region is because of pair breaking. These energy evolutions of conductance map indicate signatures of a vortex core (Fig. 4a–e), and the evolution of tunnelling spectra along line A–B in Fig. 4a clearly shows a typical spatial evolution of spectral shape across a vortex core (Fig. 4f).

To evaluate *ξ*, we analysed the zero-bias conductance data as a function of the radial distance from the vortex core centre *r* (Supplementary Fig. 3). We first extracted the zero-bias conductance *Z* as a function of mean distance *r*. Then, we fitted the *Z*(*r*) by the exponential decay function *Z*(*r*)=*Z*(∞)+*A*exp(−*r*/*ξ*), where *A* is a constant and *Z*(∞) is the normalized zero-bias conductance away from the vortex core^42^. From the fitting, we obtained *ξ*=12±1 nm (Fig. 4h), *A*=0.505±0.02 and *Z*(∞)=0.512±0.03. This *ξ* value is much larger than the *ξ*_GL_ values obtained from the upper critical field (*H*_c2_) using the formula *H*_c2_=*Φ*_0_/2*πξ*_GL_^2^ (*Φ*_0_ is the magnetic flux quanta) based on Ginzburg–Landau theory. For epitaxial LiTi_2_O_4_ thin films, the *ξ*_GL_ values estimated from macroscopic measurements are 4.1–4.7 nm (refs 22, 37). Whereas excellent agreement between *ξ* and *ξ*_GL_ values have been reported in other superconducting systems such as Fe-based superconductors^42^,^43^, the values of *ξ* obtained on the surface of LiTi_2_O_4_ are much larger than the estimated *ξ*_GL_ obtained from transport measurement techniques. The deviation of *ξ* from *ξ*_GL_ also implies that there is a difference in superconductivity between surface and bulk.

## Discussion

**Figure 5:**
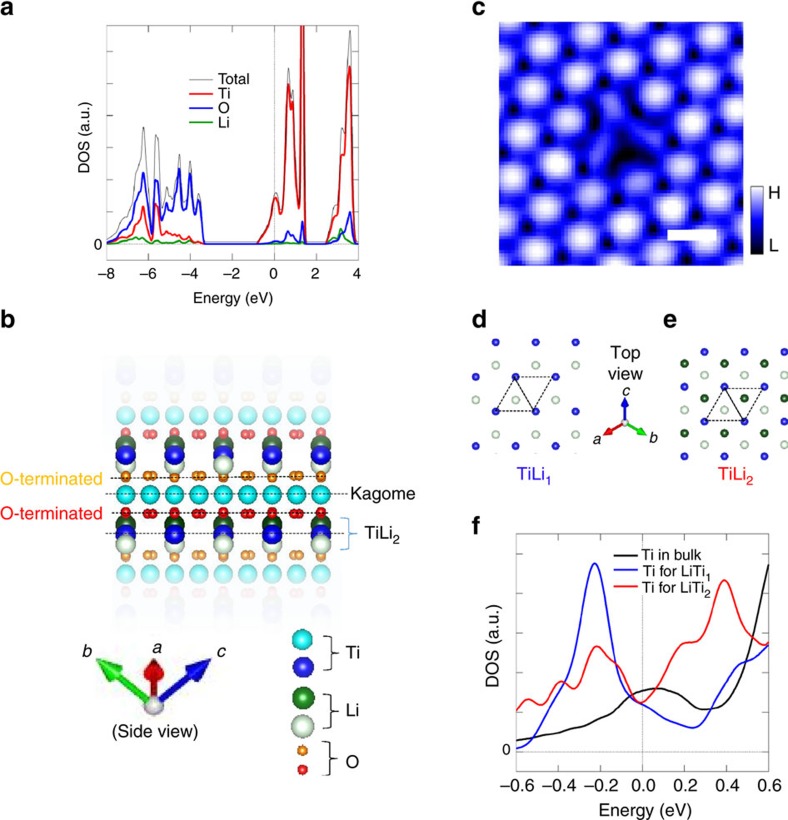
Comparison between bulk and surface electronic states based on DFT calculations. (**a**) Calculated DOS for bulk LiTi_2_O_4_. (**b**) Crystal structure with bulk continuum, together with four bulk-cut planes represented by broken lines. Vertical axis is along the (111) crystal orientation. (**c**) The topographic image of a defect on the surface (a sample-bias voltage of +30 mV, a tunnelling current of 30 pA). Scale bar, 0.6 nm (**c**). Top view of TiL_2_- (**d**) and TiLi_1_- (**e**) terminated surfaces. See **b** for the colour of the circles. (**f**) Calculated DOS for the topmost Ti atoms in TiL_2_- and TiLi_1_-terminated surfaces. DOS for bulk is shown again for clarity. Here 0 on the horizontal axes in **a**,**f** correspond to the Fermi energy.

To understand the superconductivity on the LiTi_2_O_4_(111) surface, we performed first-principles calculations. We first calculated the bulk electronic structures to understand the triangular lattice observed in the STM images. Figure 5a shows the calculated partial DOS for bulk. The Ti 3*d* states predominantly contribute near *E*_F_, and the influence of Li atoms should be negligible to the STM images. Thus, the main protrusions of the triangular lattice observed in the topographic image (indicated by blue colour in Fig. 2) correspond to Ti atoms on the surface.

To further understand the LiTi_2_O_4_(111) surface, we calculated the electronic structures of four possible bulk-cut surfaces: two-types of O-terminated, Kagome-lattice Ti-terminated, and TiLi_2_-terminated surfaces (see four dotted lines in Fig. 5b). These surfaces were optimized structurally and the electronic states of the reconstructed surfaces were investigated. Both the two O-terminated surfaces resulted in an insulating band structure (Supplementary Fig. 4a,b), which is inconsistent with the experimental metallic tunnelling spectra (Fig. 3). For the Kagome-lattice Ti-terminated surface, the simulated charge density plot (Supplementary Fig. 4c) also shows inconsistency with the experimental topographic image (Fig. 2). Consequently, neither the O-terminated nor the Kagome-lattice Ti-terminated surface reproduced the experimental results (Supplementary Fig. 4). On the other hand, TiLi_2_-terminated surface shows metallic states, and the arrangement of the protrusions and their nearest neighbour distance (0.6 nm) observed in the STM image (Fig. 2) can be explained by the framework of Ti-triangular lattice of TiLi_2_ layer.

We focus on the TiLi_2_-terminated surface, and further investigate the effect of Li-layer deficiency near surface since Li may be easily deficient during depositions process due to its high volatility. The TiLi_2_-terminated surface contains a triangular lattice of Ti atoms, and two layers of Li atoms: Li atoms displaced towards the vacuum (hereafter called higher Li layer, dark green circle in Fig. 5b) and those displaced towards the bulk (hereafter called lower Li layer, light green circle in Fig. 5b). Three possible models of surface terminations are considered: a stoichiometric TiLi_2_-terminated surface, a surface without the higher Li layer (TiLi_1_-terminated surface), and a surface without both higher and lower Li layer (TiLi_0_-terminated surface) (Fig. 5b).

On the basis of the following discussion, we could exclude TiLi_0_-terminated surface by showing that Li atoms reside at the vicinity of the topmost Ti-triangular lattice. Figure 5c shows a close-up image of the dark spots observed in the wide-area STM image (Fig. 3a). Three oval protrusions are observed, and this image indicates that a defect centre is around the middle of the three oval protrusions. Considering that the ovals are at the Ti sites, and taking into account the crystal structure of spinel system, the defects could be identified as a point-Li defect. Indeed, the dark contrast around this point-Li defect, observed at a negative sample-bias voltage of −900 meV, is consistent with hole-doping nature of Li vacancy (Fig. 2a). These results demonstrate that Li atoms reside at the vicinity of the topmost Ti-triangular lattice. Accordingly, the results exclude TiLi_0_ termination, and the surface of the films is terminated with either TiLi_2_ or TiLi_1_ structure. We note that the density of point-Li defects on the surface is <2% of Li atoms. Thus, we speculate that the impact of the presence of point-Li defects on superconductivity can be negligible^25^, unless the defects on the surfaces induce local magnetic moments.

We now compare the DOS at *E*_F_, *N*(*E*_F_), of a bulk and that of the TiLi_2_- and TiLi_1_- terminated surfaces, and reveal that both surfaces have smaller *N*(*E*_F_) than that of the bulk. Our DFT calculations for bulk show a peak structure at *E*_F_ (Fig. 5a), which is consistent with previous calculations using the linear muffin-tin orbital method^44^ and full-potential linearized augmented plane wave^45^. The simulated peak structure at *E*_F_ is also consistent with an experimental report of large normal-state electronic-specific heat, which is a measure of *N*(*E*_F_) for bulk^35^. On the TiLi_2_- (Fig. 5d) and TiLi_1_- (Fig. 5e) terminated surfaces, a broken lattice symmetry normal to the surface lifts the degeneracy of the t_2g_ orbitals and modifies the orbital states on the surface. Compared to the bulk, we observed reduction of the *N*(*E*_F_) at the topmost Ti atoms for both TiLi_2_ and TiLi_1_ (Fig. 5f). Because the smaller *N*(*E*_F_) leads to lower *T*_c_ according to the BCS theory, the calculation naively suggest suppressed superconductivity on the surface.

Based on the above discussions, we present here coherent interpretation of the experimental results. As we experimentally observed a pseudogap state (shaded red in Fig. 3a,b), which is expected to be absent in bulk, the modified superconductivity on the surface is a reasonable hypothesis. Indeed, using bulk *T*_c_ and surface *Δ*, the 2*Δ*/*k*_B_*T*_c_ value becomes anomalously small. This is puzzling since the value 3.0 is much smaller than that for a weak coupling limit of BCS superconductors (3.52). However, this puzzle can be explained by considering the presence of a non-superconducting or reduced-*T*_c_ surface layer. Moreover, the large *ξ* value can possibly be understood as the reduced *N*(*E*_F_) due to pseudogap formation on the surface. The Fermi velocity *v*_F_ is proportional to 1/*N*(*E*_F_); therefore, based on the formula *ξ* = *v*_F_/*D* derived from BCS theory, the larger *v*_F_ together with smaller *D* on a surface increases *ξ* more than that for the bulk value. Beyond the framework of the less-interacting picture, it is an interesting future subject to investigate relations between modified superconductivity, pseudogap formation and frustration effects on this (111) oriented spinel oxide surface.

In summary, we have investigated superconductivity in the atomically well-defined surface of LiTi_2_O_4_(111) thin films, using STM/STS and first-principles calculations. We provided spectroscopic evidence of modified superconductivity on the surface, originating from the formation of a pseudogap in the DOS. Our study has made an essential first step towards exploring superconducting phenomena emerging from spontaneous atomic and electronic reconstruction on surfaces, including different film orientation^46^, of superconducting cubic transition-metal oxides.

## Methods

### Samples and characterizations

We used a low-temperature STM connected with a PLD chamber. This system enables us to investigate thin film surfaces immediately after their deposition, without exposing surfaces to air. The base pressure of the PLD chamber was 5 × 10^−11^ Torr. Thin films of LiTi_2_O_4_ are grown on Nb(0.05 wt %)-doped SrTiO_3_(111) substrate using PLD with a KrF excimer laser (wavelength *λ*=248 nm). We used Li_4_Ti_5_O_12_ target for PLD film growth to compensate the Li loss during depositions. Substrates were annealed at 1,000 °C for an hour in oxygen under a partial pressure *P*_O_2__=5 × 10^−7^ Torr before film depositions. Substrates were resistively heated, and their temperatures were monitored using a pyrometer. We deposited film at a substrate temperature *T*_s_=300 °C under *P*_O_2__=5 × 10^−7^ Torr followed by post-deposition annealing at 600 °C in ultrahigh vacuum for one hour. During thin film depositions, pulse repetition and fluence were set at 2 Hz and 1 J cm^−2^, respectively. Film thickness, measured *ex situ* using a DEKTAK 3030ST mechanical profiler, was determined to be about 100 nm. The STM data shown in Fig. 1a-c were taken at liquid-nitrogen temperature (77 K). The rest of STM data were taken at liquid-helium temperature (4.2 K). We obtained differential conductance values (d*I*/d*V*) from the numerical derivatives of *I* (tunnelling current) − *V* (voltage) curves. For *ex situ* characterization, we measured X-ray diffraction pattern and temperature dependence of magnetization after *in situ* STM/STS measurements.

### First-principles calculations

For the first-principles calculations, we used DFT with the code Quantum ESPRESSO, with the generalized gradient approximation and ultra-soft pseudopotential scheme^47^,^48^,^49^,^50^,^51^. Cutoff energies for the Kohn–Sham orbitals and charge density of 32.5 and 45 Ry are imposed. The Brillouin-zone summation is evaluated using 3 × 3 × 1 and 12 × 12 × 1 *k*-point sampling for the structure optimizations and the DOS calculations, respectively. For the DOS broadening, we applied a simple Gaussian broadening method with broadening parameter of 0.005 Ry. Convergence criteria of the structure optimization are 10 × 10^−3^ for forces and 10 × 10^−4^ for energy. For the calculation of the near-surface atomic/electronic structures, we use the symmetric slab model with four unit cells along the (111) direction on each side. In the structure optimization, the topmost surface structures are initiated from bulk cuts. The atomic positions of the Ti atoms at the centre of the symmetric slab model along the (111) direction are fixed during optimization. Spin degrees of freedom and electron–electron correlations are not included in the calculation.

### Data availability

The data that support the findings of this study are available from the corresponding authors on reasonable request.

## Additional information

**How to cite this article:** Okada, Y. *et al*. Scanning tunnelling spectroscopy of superconductivity on surfaces of LiTi_2_O_4_(111) thin films. *Nat. Commun.* **8,** 15975 doi: 10.1038/ncomms15975 (2017).

**Publisher’s note:** Springer Nature remains neutral with regard to jurisdictional claims in published maps and institutional affiliations.

## Supplementary Material

Supplementary Information
