# Supplementary material for: Scanning tunnelling spectroscopy of superconductivity on surfaces of LiTi2O4(111) thin films
Source: Nat Commun. 2017 Jul 3;8:15975. doi: 10.1038/ncomms15975 (PMC5500876; doi:10.1038/ncomms15975)
Supplement: Supplementary Information [file ncomms15975-s1.pdf]

Type of file: PDF
Size of file: 0 KB
Title of file for HTML: Supplementary Information
Description: Supplementary Figures

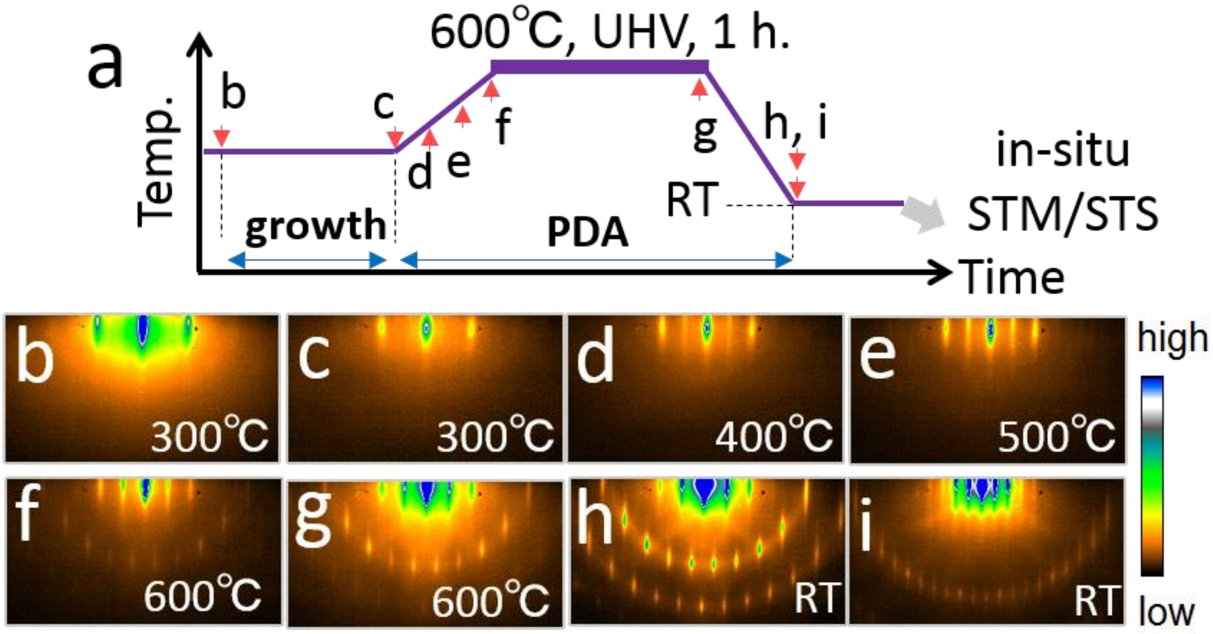

Supplementary Figure 1 | Growth and post-deposition annealing (PDA) processes. (**a**) Substrate temperature profile during thin-film growth and PDA processes. Typical ramping and cooling rates were +20°C/min and -50 °C/min, respectively. (**b-i**) Reflection high-energy electron diffraction (RHEED) patterns obtained in growth and PDA processes. Letters **b** - **i** shows the timing when the RHEED patterns were obtained. Electron beam was incident along [-110] and [-210] direction for (**b**)-(**h**) and (**i**), respectively.

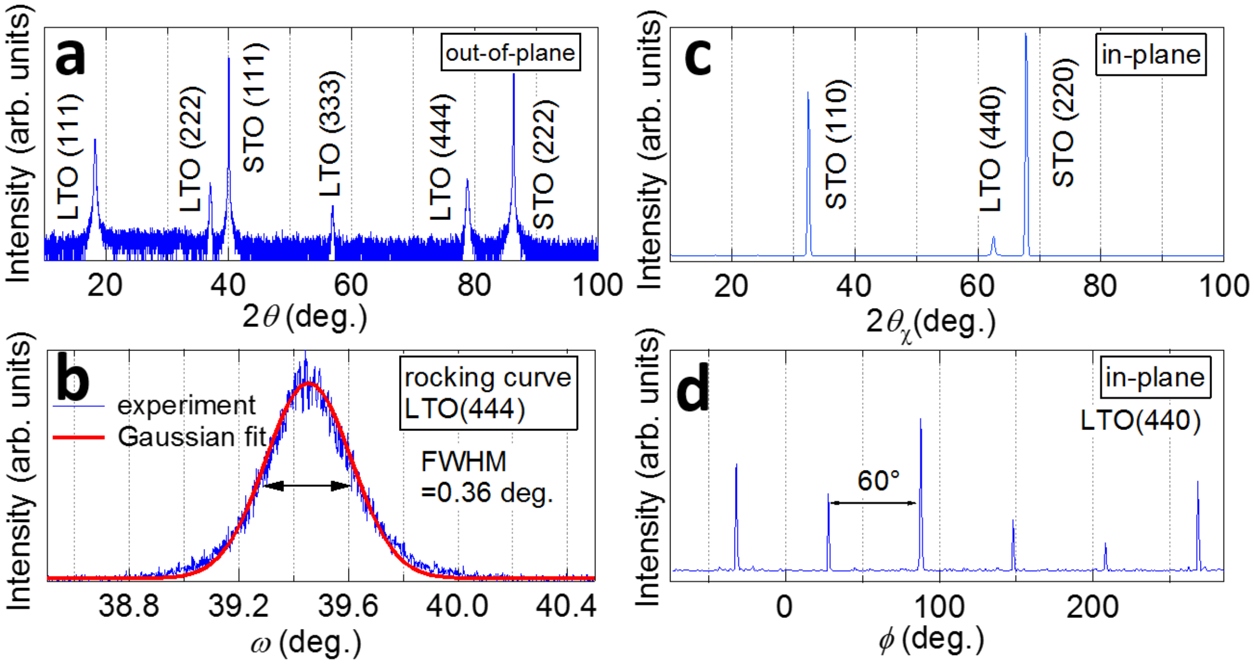

Supplementary Figure 2 | *Ex situ* characterization of films using X-ray diffraction (XRD).

(**a**) The Out-of-plane XRD pattern of a $LiTi_2O_4$ (LTO) film on a $SrTiO_3$ (STO) (111) substrate. The film was deposited at 300°C followed by the post-deposition annealing in vacuum at 600°C. (**b**) Rocking curve of the 440 peak. The full-width at half maximum (FWHM) value was ~ 0.36°. Intensities of **a** and **b** are given in logarithmic and linear scale, respectively. (**c**) and (**d**) are in-plane XRD patterns, showing that the film is epitaxially grown on the substrate.

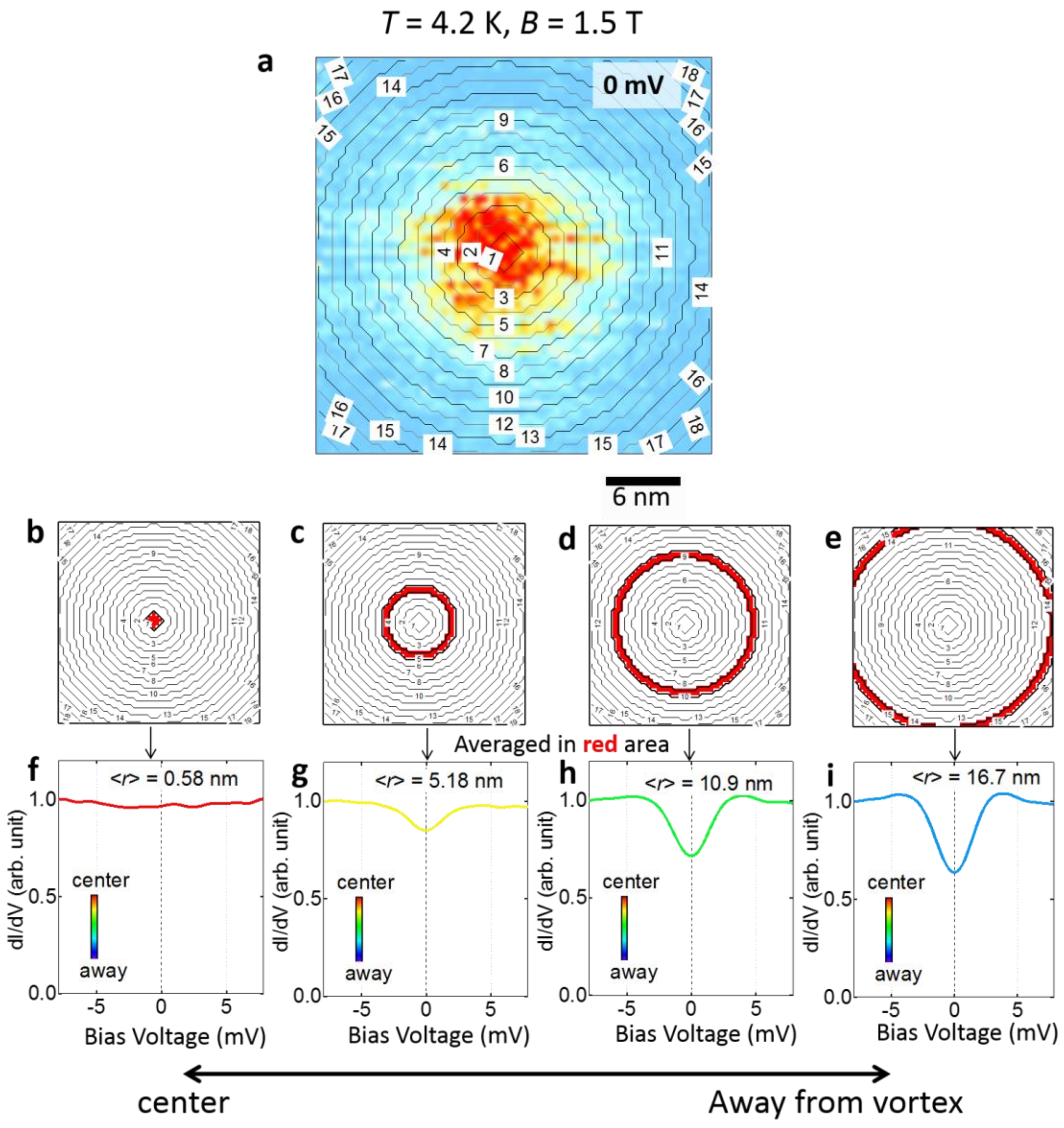

Supplementary Figure 3 | Procedures to analyze zero-bias conductance. To determine the center of a vortex, we first fit the zero-bias conductance mapping (Fig. 4c) with a 2D Gaussian. Then, using concentric circles around the vortex center, we classified the data shown in Fig. 4c into 20 regions as shown in **a**. We averaged spectra within the regions, and the series of spectra were obtained from the closest (red in Fig. 4h) to farthest (purple in Fig. 4h) from the vortex center. We show four examples of the regions (marked with red in **b-e, <*r*>** denotes an average distance from the center) together with the averaged spectra obtained within the regions (**f-i**). The data were obtained at a temperature *T* and magnetic field *B* of 4.2 K and 1.5 T, respectively.

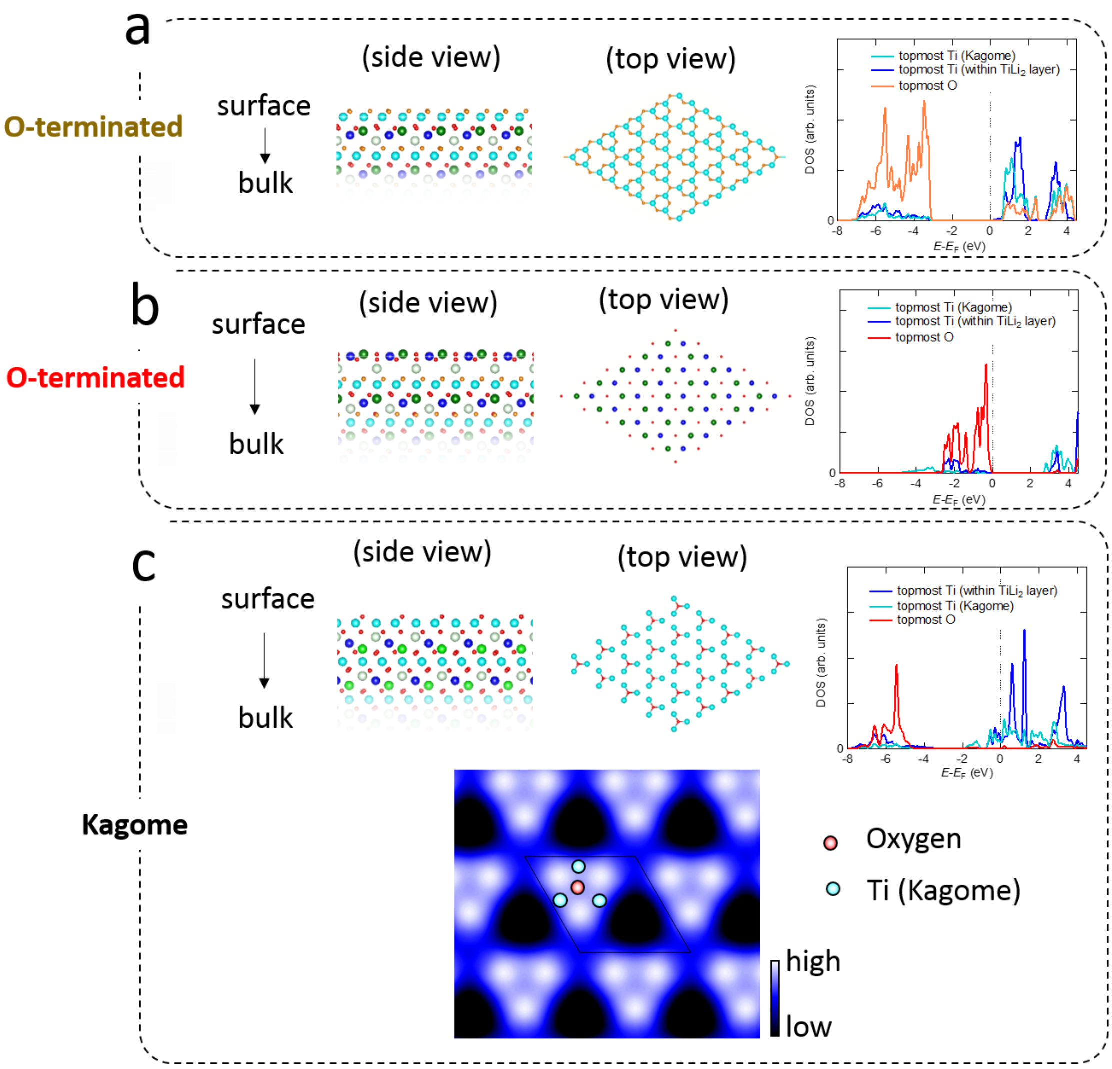

Supplementary Figure 4 | Results of density functional theory (DFT) calculations. (**a–b**) Optimized near-surface structures (left) and partial density of states (right) for two O-terminated bulk cuts in Fig. 5b. The results clearly show that these models exhibit insulating electronic structure on the surface, which is inconsistent with experimental metallic tunneling spectra (Fig. 3). (**c**) Optimized near-surface structures (upper left) and partial density of states (upper right) for Kagome-lattice Ti-terminated surfaces in Fig. 4a. The surface atomic structures reconstruct, and oxygen atoms reside at the center of three Ti atoms. The bottom image shows a Tersoff–Hamann simulated charge density plot at E - E_F = 0 eV at 0.2 nm above the topmost oxygen atom. The image does not reproduce the experimental results shown in Fig. 2.
